# Ecological Momentary Assessments and Passive Sensing in the Prediction of Short-Term Suicidal Ideation in Young Adults

**DOI:** 10.1001/jamanetworkopen.2023.28005

**Published:** 2023-08-08

**Authors:** Ewa K. Czyz, Cheryl A. King, Nadia Al-Dajani, Lauren Zimmermann, Victor Hong, Inbal Nahum-Shani

**Affiliations:** 1 Department of Psychiatry, University of Michigan, Ann Arbor; 2 Department of Psychology, University of Michigan, Ann Arbor; 3 Now with Department of Psychological and Brain Sciences, University of Louisville, Louisville, Kentucky; 4 Institute for Social Research, University of Michigan, Ann Arbor

## Abstract

**Importance:**

Advancements in technology, including mobile-based ecological momentary assessments (EMAs) and passive sensing, have immense potential to identify short-term suicide risk. However, the extent to which EMA and passive data, particularly in combination, have utility in detecting short-term risk in everyday life remains poorly understood.

**Objective:**

To examine whether and what combinations of self-reported EMA and sensor-based assessments identify next-day suicidal ideation.

**Design, Setting, and Participants:**

In this intensive longitudinal prognostic study, participants completed EMAs 4 times daily and wore a sensor wristband (Fitbit Charge 3) for 8 weeks. Multilevel machine learning methods, including penalized generalized estimating equations and classification and regression trees (CARTs) with repeated 5-fold cross-validation, were used to optimize prediction of next-day suicidal ideation based on time-varying features from EMAs (affective, cognitive, behavioral risk factors) and sensor data (sleep, activity, heart rate). Young adult patients who visited an emergency department with recent suicidal ideation and/or suicide attempt were recruited. Identified via electronic health record screening, eligible individuals were contacted remotely to complete enrollment procedures. Participants (aged 18 to 25 years) completed 14 708 EMA observations (64.4% adherence) and wore a sensor wristband approximately half the time (55.6% adherence). Data were collected between June 2020 and July 2021. Statistical analysis was performed from January to March 2023.

**Main Outcomes and Measures:**

The outcome was presence of next-day suicidal ideation.

**Results:**

Among 102 enrolled participants, 83 (81.4%) were female; 6 (5.9%) were Asian, 5 (4.9%) were Black or African American, 9 (8.8%) were more than 1 race, and 76 (74.5%) were White; mean (SD) age was 20.9 (2.1) years. The best-performing model incorporated features from EMAs and showed good predictive accuracy (mean [SE] cross-validated area under the receiver operating characteristic curve [AUC], 0.84 [0.02]), whereas the model that incorporated features from sensor data alone showed poor prediction (mean [SE] cross-validated AUC, 0.56 [0.02]). Sensor-based features did not improve prediction when combined with EMAs. Suicidal ideation-related features were the strongest predictors of next-day ideation. When suicidal ideation features were excluded, an alternative EMA model had acceptable predictive accuracy (mean [SE] cross-validated AUC, 0.76 [0.02]). Both EMA models included features at different timescales reflecting within-day, end-of-day, and time-varying cumulative effects.

**Conclusions and Relevance:**

In this prognostic study, self-reported risk factors showed utility in identifying near-term suicidal thoughts. Best-performing models required self-reported information, derived from EMAs, whereas sensor-based data had negligible predictive accuracy. These results may have implications for developing decision algorithms identifying near-term suicidal thoughts to guide risk monitoring and intervention delivery in everyday life.

## Key Points

**Question:**

Are real-time assessments of self-reported experiences and wearable sensor data useful in predicting near-term suicidal thoughts?

**Findings:**

In this prognostic study with 102 participants, young adults completed 4 daily mobile-based surveys and wore a sensor wristband for 8 weeks after an emergency department visit. Models incorporating self-reported information from mobile-based assessments achieved good prediction of next-day suicidal ideation, whereas sensor-based assessments, alone or in combination with self-reported data, had poor prediction performance.

**Meaning:**

These results suggest that self-reported risk factors have utility in identifying near-term suicidal thoughts, with implications for decision algorithms guiding risk monitoring and interventions during high-risk care transitions.

## Teaser

This prognostic study investigates whether various combinations of self-reported ecological momentary assessments (EMAs) and sensor-based assessments are capable of predicting next-day suicidal ideation.

## Introduction

Preventing suicide mortality, the second leading cause of death in adolescents and young adults,^1^ as well as reducing extreme pain and distress associated with suicidal thoughts and nonlethal attempts remain an urgent public health priority. Although many factors can increase risk for suicidal ideation and behavior, postacute care transitions, such as following emergency department (ED) or inpatient services, are linked to increased vulnerability.^2,3^ Identifying heightened suicide risk during high-risk periods will likely require approaches that are sensitive to its complex and time-varying nature. Prior research shows that single risk factors assessed weeks or months apart have a weak association with suicidal thoughts and behavior.^4,5^ Moreover, suicidal thoughts are far from static and fluctuate considerably within short intervals, from one day to the next or several hours apart.^6,7,8^ In line with national research priorities calling for improved understanding of short-term suicide risk,^9^ examining what combinations of risk factors may signal impending (eg, within days or hours) suicidal thoughts is an important step toward informing short-term risk detection and intervention targets.

Real-time assessments leveraging mobile and wearable sensor technologies offer unique opportunities to identify short-term suicide risk. Mobile-based ecological momentary assessments (EMAs), which rely on repeated measurement of self-reported experiences in daily life, are increasingly applied in suicide prevention research, with the majority of studies to date focusing on delineating proximal relationship between individual risk factors and suicidal ideation.^10,11,12^ Given the complexity of suicide risk, additional research is needed to examine how time-varying affective, cognitive, and interpersonal experiences may come together, beyond individual associations, to confer short-term risk. Another key weakness of existing EMA studies lies in their overreliance on self-reporting, which may increase respondent burden and decreased engagement over time.^13,14^ Passive sensing can be used to collect objective data without requiring direct input from individuals, thus holding great potential in identifying suicidal ideation and behavior.^15,16^ For example, passive data could be used to unobtrusively detect processes that map onto suicide risk factors, such as sleep, distress captured by heart rate, social interaction patterns, or physical activity as proxy of withdrawal or mood.^15,17^ However, empirical evidence supporting the utility of passive data in predicting short-term suicidal ideation or behavior remains limited.

To date, studies integrating EMA and passive sensing to identify short-term suicide risk have primarily focused on the association between sleep disturbance and suicidal ideation, with mixed results. While sensor-based assessments of sleep were shown to predict proximal (next-day) suicidal ideation by some,^18^ others highlighted either lack of next-day association^19^ or an indirect link.^20^ Moreover, recent studies incorporating sensor-based assessment of sleep^19^ and physiological distress^21^ point to passive data having a relatively weaker association with near-term suicidal thoughts compared with self-reported information from EMAs. This suggests that the association between passive data and suicide risk-related outcomes may be more nuanced and warrants further investigation.

In this study of young adults with a recent ED visit, we expand on existing literature to examine the utility of sensor-based and EMA data in predicting short-term suicidal thoughts. Specifically, we applied machine learning models incorporating time-varying predictors from EMAs and a wearable sensor, separately and then in combination, to identify next-day suicidal ideation. We initially considered different theoretically informed and clinically informed predictors derived from EMAs (affective, cognitive, and interpersonal domains) and from sensor-based assessments, measured with a commercial sensor wristband, that map onto relevant risk factors (distress captured with heart rate, sleep disturbance, and physical activity as proxy of low mood or withdrawal).^15,17^ Investigating which combination of EMA and passive features are most promising in predicting proximal suicidal thoughts in everyday life is a critical step in the development of timely interventions that can address elevations in suicidal thoughts (ie, when these risk states are detected). To our knowledge, no prior studies have examined what combinations of EMA and passive predictors are optimal in identifying short-term suicidal ideation during high-risk care transitions.

## Methods

### Participants and Procedures

Participants were ED patients (aged 18 to 25 years) recruited between June 2020 and May 2021. Electronic health records (EHRs) were screened for last-month suicide attempt and/or last-week suicidal ideation based on the Columbia-Suicide-Severity Rating Scale (C-SSRS^22^), administered routinely, and supplemented with EHR data. Exclusion criteria included altered mental state (eg, acute psychosis, mania), cognitive impairment, transfer to jail or police custody, or other reasons preventing contact (eg, no contact information). Of the 911 individuals who had EHRs screened, 429 individuals (47.1%) met initial eligibility and were contacted by email and/or phone. Of these, 289 did not respond to contacts (67.4%), 4 (0.9%) responded but could not be enrolled (no cell phone, participating in another study), 16 (3.7%) declined participation, and 120 provided study consent (28.0%). A total of 110 participants were enrolled, with the analytic sample restricted to the 102 who continued in the study (3 withdrew) and had consecutive follow-up data (1 provided no data, 4 had no consecutive observations). To describe sample characteristics, self-reported race and ethnicity data were obtained.

The study was approved by the University of Michigan institutional review board and followed the Transparent Reporting of a Multivariable Prediction Model for Individual Prognosis or Diagnosis (TRIPOD) reporting guideline.^23^ Study consent and enrollment procedures, including enrollment in MetricWire and Fitbit mobile applications, were completed by telephone. Participants were subsequently mailed a sensor wristband (Fitbit Charge 3). Data collection began after all enrollment procedures were completed, a mean (SD) of 8.75 (3.71) days after ED visit. For 8 weeks, participants completed 4 EMAs between 9:30am and 9:30pm, randomly sampled within 4 time blocks (morning, early afternoon, late afternoon, evening), and were compensated up to $304 based on adherence. Participants were also asked to wear the study-provided sensor wristband, with weekly automated reminders to wear and charge the device. EMA completion across 102 participants was 64.4% (14 708 out of 22 848 possible). Sensor wristband adherence was 55.6% (a mean of 44 832.33 minutes of use out of 80 640.00 total minutes possible per participant) for 98 participants with any sensor wristband data (1 participant did not receive the mailed sensor wristband, 3 did not wear the device).

### Measures

#### Outcome

Participants indicated whether they experienced suicidal ideation and, if present, duration of ideation. The question was modeled after the C-SSRS items.^22^ Response options matched the observation period such that, at each EMA, participants responded in reference to the last hour (observation-level ideation) and, as part of the last EMA of the day, in reference to the entire day (daily-level ideation). A binary suicidal ideation indicator was obtained based on both observation-level and daily-level responses to capture any reported suicidal ideation each day.

#### EMA Predictors

Self-reported constructs were assessed with single-item measures based on existing scales, adapted in prior EMA research to reduce response burden.^24^ Participants responded to items in reference to the last hour (at each EMA occasion) and in reference to the entire day (evening EMA) or prior day (morning EMA). Additional information is provided in eMethods 1 in Supplement 1. *Hopelessness* was measured with an item modeled after the Brief Hopelessness Scale.^25^ *Connectedness* and *burdensomeness* were each measured using items from the Interpersonal Needs Questionnaire (INQ).^26^ *Agitation* was measured with an item modeled after the Brief Agitation Measure.^27^ *Worry* and *rumination* were each assessed with an item derived from prior EMA studies of rumination and worry.^28^ *Self-efficacy to refrain from suicidal action* was assessed with an item from the Self-Assessed Expectations of Suicide Risk Scale.^29^ *Positive* and *negative affect* was measured with 5 items derived from the PANAS.^30,31^ *Thoughts about death frequency* and *duration* together with *suicidal ideation frequency* and *duration* were measured with items based on the C-SSRS^22^; when thoughts of suicide were present, a follow-up item assessed ideation *intensity*. Based on items from prior EMA studies of suicide risk,^32^ we assessed presence of *nonsuicidal self-injury (NSSI)* and extent of engaging in *coping behavior* incorporating cognitive, noncognitive (relaxation, distraction), and support-seeking strategies. *Presence of a negative interpersonal event* (eg, serious argument, breakup) was based on prior studies of interpersonal suicide warning signs.^33,34^ *Alcohol consumption*, in standard drinks from none to 10 or greater, was measured with an item from a prior daily study of substance use^35^ and was categorized from none to 5 or more. *Sleep quality* was assessed with an item from the PROMIS sleep measure.^36^

#### Sensor-Based Predictors

We collated data from participants’ sensor wristbands using Fitabase, a research platform that enables data transfer. The following daily-level metrics were obtained: *Resting heart rate*, *Total steps*, and *Sleep duration* (total minutes asleep). For a metric of daily-level *Heart rate variability*, we derived the root mean squared successive differences (RMSSD) of R-R interval estimates based on raw 1-minute heart rate. The average of the successive differences was computed over 5-minute intervals^37^ and subsequently averaged to calculate daily-level RMSSD. Additional details are in eMethods 2 in Supplement 1.

#### Predictor Preparation

For each day, we constructed a set of features derived from constructs assessed multiple times with EMAs (ie, across within-day EMAs) as well as once-daily (evening EMA or morning EMA), in addition to features from sensor-based assessments. We calculated the following EMA and sensor-based features in a manner that preserved temporality of data, capturing information available up to day *t*: (1) time-varying cumulative person-specific mean for each day *t* (ie, within-person sum of EMA or sensor-based observations up to and including day *t*, divided by the number of available observations up to and including *t*); (2) deviation of day-level *t* observation from the time-varying cumulative person-specific mean; (3) previous day *t* observation; and (4) maximum within-day observation for each day *t*. Features were entered into prediction models.

### Statistical Analysis

To address the primary objective of predicting next-day suicidal ideation, we applied mixed-effects classification and regression trees (CARTs) using features from EMAs, sensor-based assessments, and their combination. Features were first selected via penalized generalized estimating equations (PGEE)^38^ using PGEE package in R.^39^ We used this regularization method because it is suitable for the longitudinal data and multiple features available.^38^ Features with nonzero coefficients (ie, correlated with the outcome) were included in CART models. Using the glmertree R package, we used a generalized linear mixed-effects model tree (GLMM tree) algorithm,^40^ which flexibly captures interactions and nonlinearity among the features, while accounting for the longitudinal (ie, nested) structure of the data. This approach iteratively estimates (1) the tree (ie, fixed effects) given an offset of random effects and (2) the random effects given the structure of the tree. Models were built without missing data imputation. However, all models included a missingness indicator for each day (proportion of completed EMAs, proportion of minutes the sensor wristband was worn). We also included time indicators (day in the study, day of week). eMethods 3 and 4 in Supplement 1 provide further details on the data analytic approach. To assess model performance, we performed stratified blocked 5-fold cross-validation with 10 repetitions, using the groupdata2 R package.^41^ The *k*-fold cross-validated procedure partitions the sample into *k* folds to test model performance on an independent data set, by reserving *k* − 1 folds for training and leaving the *k*th fold for testing for all folds *k* = 1, …, *K*. In this application of stratified blocked cross-validation, participants served as blocks (observations from a given participant are kept within the same fold), with individuals endorsing high and low proportion of ideation days kept balanced across the folds. As the primary prediction performance metric, we examined mean with corresponding standard error of cross-validated area under the receiver operating characteristic curve (AUC), which ranges from 0.5 (chance predictive accuracy) to 1.0 (perfect predictive accuracy). To compute variable importance from the mixed-effects CART, we used a variance-based method^42^ using the vip package in R.^43^ Analyses were performed in R version 4.2.2 (R Project for Statistical Computing) from January to March 2023.

## Results

### Baseline Characteristics

Among 102 enrolled participants, 83 (81.4%) were female; 6 (5.9%) were Asian, 5 (4.9%) were Black or African American, 9 (8.8%) were more than 1 race, and 76 (74.5%) were White; the mean (SD) age was 20.9 (2.1) years (Table 1). At index ED visit, all participants reported suicidal ideation, with mean (SD) past-week suicidal ideation being 3.08 (1.17) (scale range: 0-5, where 0 corresponds to no ideation and 5 to ideation with intent and plan); 54 participants (52.9%) reported history of suicide attempts, and 5 (4.9%) attempted suicide in the last month. Obtained via EHR review, the most frequent diagnosis at index ED visit was a depressive disorder, including major depressive disorder or unspecified depressive disorder (75 participants [73.5%]), followed by an anxiety-related disorder, including panic disorder, generalized anxiety disorder, or unspecified anxiety disorder (32 participants [31.4%]).

**Table 1.** Sample Characteristics

Baseline characteristics	Enrolled participants (N = 102), No. (%)
Age, mean (SD), y	20.9 (2.1)
Sex, No. (%)	
Male	19 (18.6)
Female	83 (81.4)
Gender self-reported, No. (%)	
Male	16 (15.7)
Female	68 (66.6)
Female-to-male transgender	7 (6.9)
Male-to-female transgender	1 (1.0)
Genderqueer/gender nonconforming	7 (6.9)
Other^a^	3 (2.9)
Race self-reported, No. (%)	
American Indian or Alaska Native	2 (2.0)
Asian	6 (5.9)
Black or African American	5 (4.9)
More than 1 race	9 (8.8)
White	76 (74.5)
Other^b^	3 (2.9)
Unknown	1 (1.0)
Ethnicity self-reported, No. (%)	
Hispanic	11 (10.8)
Not Hispanic or Latino	89 (87.2)
Unknown	2 (2.0)
Diagnoses, No. (%)^c^	
Depressive disorder (major depressive disorder, unspecified depressive disorder)	75 (73.5)
Unspecified bipolar disorder	2 (2.0)
Unspecified mood disorder	2 (2.0)
Anxiety disorder (panic disorder, generalized anxiety disorder, unspecified anxiety disorder)	32 (31.4)
Obsessive compulsive disorder	2 (2.0)
Posttraumatic stress disorder	1 (1.0)
Unspecified trauma- and stressor-related disorder	10 (9.8)
Adjustment disorder	3 (2.9)
Substance use disorder (cannabis- and/or alcohol-related)	9 (8.8)
Unspecified feeding or eating disorder	3 (2.9)

^a^ Other gender reflects the following: questioning and nonbinary.

^b^ Other race includes self-reporting of other.

^c^ Some participants had more than 1 diagnosis.

### Follow-Up Descriptive Statistics

Over the 8-week follow-up period, suicidal ideation was reported on 2197 EMA occasions (14.9%) by 89 participants (87.3%), with an additional 202 ideation disclosures reported in reference to the entire day (last survey of the day). Thus, any suicidal ideation was reported on 1346 observation days (33.4%) by 90 participants (88.2%). eFigure 1 in Supplement 1 shows individual time series plots of suicidal ideation. Table 2 shows the means, standard deviations, and interclass correlations (ICCs) for EMA and sensor-based features, with PGEE-selected features that were entered into CART models marked with a footnote citation.

**Table 2.** Descriptive Statistics for EMA and Passive Predictors

Variables (range)^a^	Mean (SD)	ICC	Derived features
EMAs (4/d)			
Happy (1-5)	2.56 (1.00)	0.57	Happy^b^ (avg), CM happy, CH happy, max happy
Miserable (1-5)	1.78 (0.91)	0.50	Miserable (avg), CM miserable, CH miserable, max miserable
Angry (1-5)	1.48 (0.74)	0.53	Angry (avg), CM angry,^b^ CH angry, max angry^b^
Nervous (1-5)	2.44 (1.09)	0.61	Nervous (avg), CM nervous, CH nervous, max nervous
Sad (1-5)	2.05 (1.02)	0.53	Sad (avg), CM sad, CH sad, max sad^b^
Rumination (1-7)	3.04 (1.78)	0.59	Rumination^b^ (avg), CM rumination,^b^ CH rumination, max rumination^b^
Worry (1-7)	3.03 (1.74)	0.59	Worry^b^ (avg), CM worry,^b^ CH worry, max worry^b^
Agitation (1-7)	2.12 (1.46)	0.56	Agitation (avg), CM agitation,^b^ CH agitation, max agitation^b^
Hopelessness (1-4)	2.04 (0.84)	0.73	Hopelessness (avg), CM hopelessness,^b^ CH hopelessness, max hopelessness
Burden (1-7)	2.30 (1.66)	0.71	Burden^b^ (avg), CM burden, CH burden, max burden^b^
Close to others (1-7)	3.69 (1.65)	0.59	Close to others (avg), CM close to others, CH close to others, max close to others^b^
Self-efficacy (0-10)	8.66 (1.95)	0.72	Self-efficacy^b^ (avg), CM self-efficacy^b^, CH self-efficacy^b^, max self-efficacy^b^
Duration of death thoughts (0-4)	0.36 (0.69)	0.48	Death thoughts duration^b^ (avg), CM death thoughts duration, CH death thoughts duration, max death thoughts duration
Duration of SI (0-4)	0.25 (0.56)	0.49	SI duration (avg), CM SI duration, CH SI duration, max SI duration
Intensity of SI (0-5)	0.32 (0.71)	0.48	SI intensity (avg), CM SI intensity, CH SI intensity, max SI intensity
Evening EMA survey			
Frequency of death thoughts (0-4)	0.70 (1.04)	0.50	Death thoughts frequency, CM death thoughts frequency, CH death thoughts frequency
Frequency of SI (0-4)	0.48 (0.89)	0.46	SI frequency,^b^ CM SI frequency,^b^ CH SI frequency^b^
Intensity of SI (0-5)	0.55 (1.04)	0.37	SI intensity, CM SI intensity, CH SI intensity
Negative relationship events, No. (%)	554 (17.7)	0.34	Negative relationship events, CM negative relationship events, CH negative relationship events
Coping (0-6)^c^	2.59 (1.84)	0.48	Coping,^b^ CM Coping,^b^ CH Coping^b^
Morning EMA survey			
Sleep quality (0-4)	2.13 (1.01)	0.30	Sleep quality, CM Sleep quality, CH sleep quality
NSSI presence, No. (%)	100 (3.2)	0.67	NSSI, CM NSSI, CH NSSI
Alcohol in standard drinks, No. (%)			
0	1927 (84.4)	0.24	Alcohol, CM alcohol, CH alcohol
1-2	201 (8.8)		
3-4	101 (4.4)		
≥5	54 (2.4)		
Sensor wristband (daily)			
Resting heart rate (beats per minute)	70.23 (10.60)	0.90	Resting heart rate,^b^ CM resting heart rate,^b^ CH resting heart rate^b^
RMSSD in milliseconds	35.07 (9.55)	0.77	RMSSD,^b^ CM RMSSD,^b^ CH RMSSD^b^
Activity in steps	6309.55 (4725.48)	0.44	Total steps, CM total steps, CH total steps
Sleep duration, min	487.18 (144.76)	0.36	Minutes asleep, CM minutes asleep,^b^ CH minutes asleep

Abbreviations: avg, average score across within-day EMAs; CH, change from cumulative mean; CM, time-varying cumulative mean; EMA, ecological momentary assessment; ICC, intraclass correlation; max, maximum score from within-day EMAs; NSSI, nonsuicidal self-injury; RMSSD, root mean square of successive difference from heart rate; SI, suicidal ideation.

^a^ For each variable range listed, higher numbers indicate higher value of the specified variable (ie, higher number for *happy* means more happiness).

^b^ Features were selected for further inclusion in the mixed-effects CART models.

^c^ Coping reflects the sum of 3 coping types: reframe, talk, and distract.

### Model Performance

**Figure 1.**
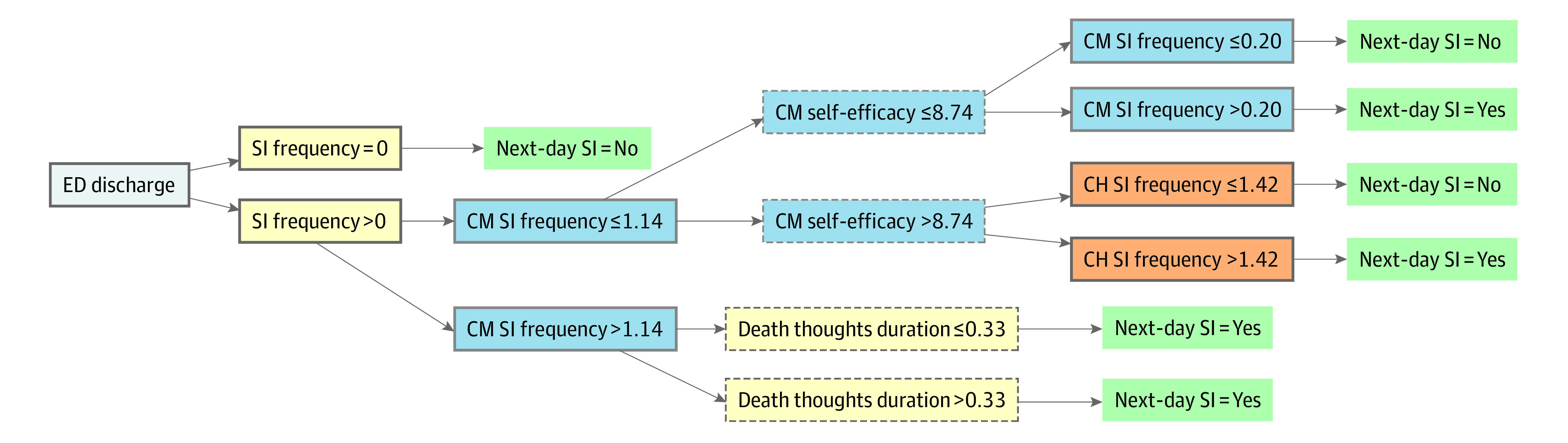
Decision Tree Predicting Next-Day Suicidal Ideation (SI) (N = 3126 Observations) Feature types are marked as follows: yellow indicates previous-day observation; (2) blue indicates cumulative mean (CM); (3) orange indicates change from cumulative mean (CH). Features from within-day observations have a dashed node border, and features from end-of-day observation have a solid node border. ED indicates emergency department.

Table 3 shows the performance of mixed-effects CARTs. The mean (SE) cross-validated AUCs were 0.84 (0.02) for EMAs, 0.56 (0.02) for sensor-based assessments, and 0.84 (0.02) for their combined features. Thus, sensor-based assessments did not improve prediction beyond EMAs. In sensitivity analyses, when replacing missing values across sensor wristband features with a large arbitrary value^44^ to ensure congruent sample sizes with EMA models, mean (SE) results were similar for sensor-based (AUC, 0.53 [0.02]) and combined (AUC, 0.84 [0.02]) models. Figure 1 displays the tree structure from the best-performing (EMA only) model. Suicidal ideation-related features, across different time scales (eg, prior-day observation; time-varying cumulative mean, change), appeared in multiple nodes, indicating strong relevance to predicting next-day suicidal thoughts. eFigures 2 and 3 in Supplement 1 additionally show relative variable importance.

**Table 3.** Model Performance^a^

Metric	EMA only (n = 3126 observations)		Passive only (n = 2177 observations), passive features	Combined EMA and passive (n = 1804 observations)	
	With SI features	Without SI features		With SI features	Without SI features
AUC (SE) [95% CI]	0.84 (0.02) [0.80-0.88]	0.76 (0.02) [0.72-0.80]	0.56 (0.02) [0.52-0.60]	0.84 (0.02) [0.80-0.88]	0.75 (0.03) [0.69-0.81]
Sensitivity (SE) [95% CI]	0.77 (0.03) [0.71-0.83]	0.70 (0.03) [0.64-0.76]	0.59 (0.03) [0.53-0.65]	0.79 (0.04) [0.71-0.87]	0.68 (0.04) [0.60-0.76]
Specificity (SE) [95% CI]	0.80 (0.02) [0.76-0.84]	0.75 (0.02) [0.71-0.79]	0.50 (0.02) [0.46-0.54]	0.80 (0.03) [0.74-0.86]	0.78 (0.03) [0.72-0.84]

Abbreviations: AUC, area under the receiver operating characteristic curve; EMA, ecological momentary assessment; SI, suicidal ideation.

^a^ This table shows 5-fold cross-validated with 10 repetitions mean metrics. Performance did not improve when replacing missing values in passive features with arbitrary positive value to have a similar sample size of 3126 observations (mean [SE] AUC for passive-only model: 0.53 [0.02]; 95% CI, 0.49-0.57; mean [SD] AUC for EMA and passive model: 0.84 [0.02]; 95% CI, 0.80-0.88).

**Figure 2.**
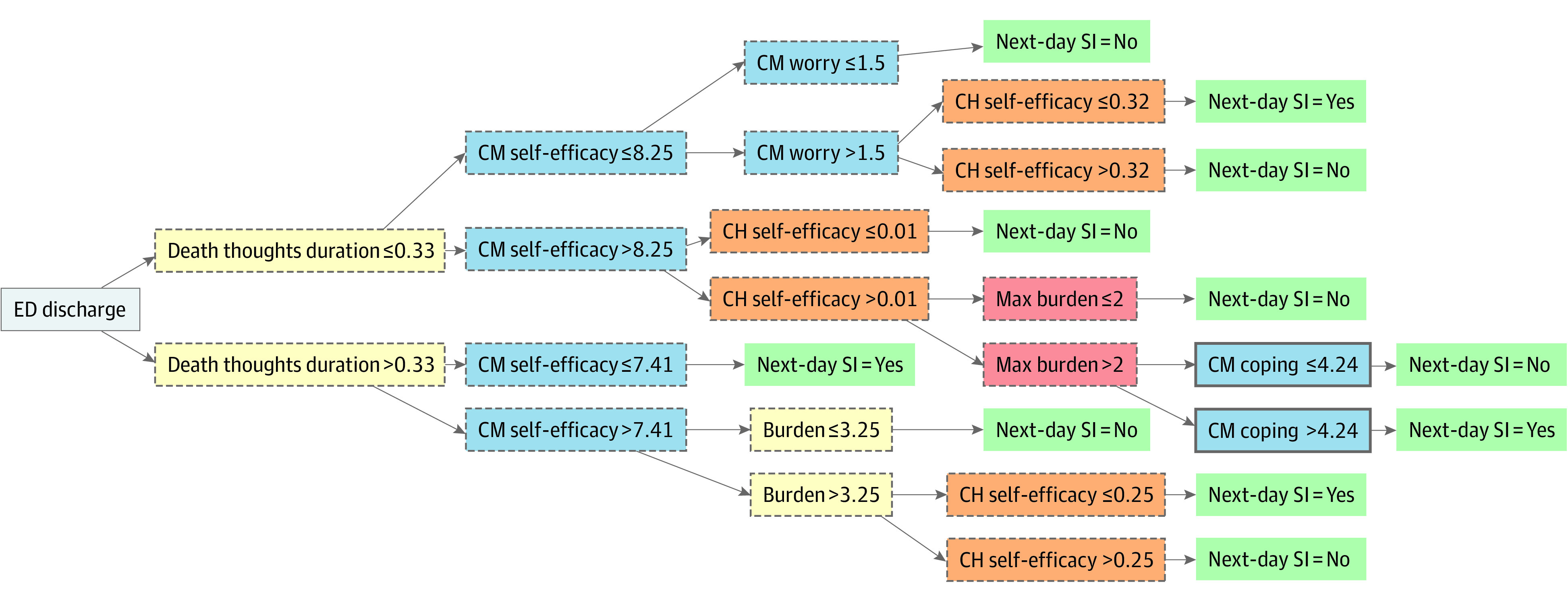
Decision Tree Predicting Next-Day Suicidal Ideation (SI) Without SI-Related Predictors (N = 3126 Observations) Feature types are marked as follows: yellow indicates previous-day observation; (2) blue indicates cumulative mean (CM); (3) orange indicates change from cumulative mean (CH); (4) red indicates maximum (max). Features from within-day observations have a dashed node border, and features from end-of-day observation have a solid node border. ED indicates emergency department.

Additionally, we assessed model performance without features explicitly asking about thoughts of killing self. eFigure 4 in Supplement 1 displays relative variable importance. As shown in Table 3 (without suicidal ideation features), the corresponding mean (SE) cross-validated AUCs were 0.76 (0.02) for the EMA model and 0.75 (0.03) for the combined model. As passive data did not improve performance, Figure 2 shows the tree structure for the EMA model without items related to suicidal thoughts.

## Discussion

In this prognostic study of young adults, we applied machine learning methods to optimize prediction of near-term suicidal ideation following an ED visit. The goal was to examine whether and what combinations of self-reported EMA and unobtrusive sensor-based data identify next-day suicidal thoughts. Our main findings were that passive sensing data did not meaningfully contribute to predicting next-day suicidal thoughts, that strongest proximal predictors included self-reported suicidal thoughts, and that predictive performance was driven by near-term experiences that accumulate and those representing shifts in functioning.

Models with EMA features had best predictive accuracy (mean [SE] AUC of 0.84 [0.02]), whereas sensor-based data, whether alone or in combination with EMAs, showed poor prediction. It is important to emphasize that sensor-based assessments included indices of physiological arousal (heart rate) and regulation (activity and sleep), and it is possible that other passive data (eg, geolocation, communication patterns) may have yielded different results. Relatedly, it may be that constructs underlying these objective markers were relatively less important in predicting next-day ideation, as the goal was to identify features that optimize prediction of short-term suicidal thoughts. For example, neither sensor-based nor self-reported sleep were retained in selected models. Nevertheless, these results are consistent with recent studies integrating passive sensing suggesting weak associations with suicide risk-related outcomes, particularly over and above self-reported metrics.^19,21,45,46^ While more streamlined, reliance on self-report alone may require relatively more attention to engagement, particularly if EMA monitoring is used more frequently or over extended periods.

The strongest predictors of next-day suicidal ideation were related to recent suicidal ideation, with key features reflecting end-of-day suicidal thoughts, time-varying cumulative suicidal thoughts, and change in suicidal thinking. The finding that suicidal ideation is a robust predictor of subsequent ideation in the near-term may not be surprising given its close relevance to the outcome and is consistent with other intensive longitudinal studies.^6,8,47^ Our results additionally highlight that near-term suicidal thoughts can be adequately predicted when excluding features measuring thoughts of killing self. While predictive accuracy for this alternative model was relatively lower (mean [SE] AUC of 0.76 [0.02] vs 0.84 [0.02]), foregoing direct queries about suicidal thoughts may be more practical in some contexts or offer an acceptable option if there are concerns about disclosures.^48,49^ Importantly, results from either model (prediction algorithm) could be used to inform just-in-time adaptive interventions (JITAIs)^50,51^ which use decision rules to guide when real-time support should be provided. Understanding the conditions in which individuals are at increased risk for experiencing near-term suicidal thoughts is an important step in developing JITAIs that deliver support to prevent or ameliorate the risk.^52^

In the current study, it was also notable that most important predictors incorporated different time scales, including end-of-day ratings, within-day averages, highest within-day scores (maximum), person-specific cumulative means, and deviations from cumulative means. The fact that prediction was driven by proximal experiences as well as their cumulative effect (time-varying cumulative means) is consistent with a study of high-risk adolescents^24^ and further highlights the importance of attending to how time-varying experiences not only change but also accumulate in the near-term to give rise to suicidal ideation in everyday life (eg, enduring distress over prior days could be further exacerbated by an acute experience). Future research is needed to examine the benefits of modeling more rapid or extreme shifts in functioning together with dynamic experiences spanning over days prior. While much research to date has focused on the former, capturing real-time experiences across a spectrum of timescales could offer additional insights into how the risk for suicide is shaped in everyday life.

### Limitations

This study had limitations. Generalizability of results is limited by the sample being composed of primarily female and White participants. Sensor-based assessments for sleep, activity, and heart rate were measured with emerging wearable sensor technology that may introduce more error than criterion standard laboratory assessments^53,54,55^; however, early evidence suggests these tools can produce meaningful data related to different mental health outcomes.^56,57^ Nevertheless, this is an important consideration as technology is improving and given growing interest in wearable sensors with the practical advantage of larger-scale use. While our focus is on time-varying predictors, future extensions could investigate initial clinical and demographic data as well as optimal frequency of assessments to balance practical concerns (eg, response burden) and predictive accuracy. Furthermore, the extent to which real-time data predict suicide attempts represents an important future direction. We selected PGEE and multilevel CART over alternative machine learning methods to maximize interpretability; however, future studies could apply different machine learning strategies to identify near-term risk. While we used repeated 5-fold cross-validation, an even more rigorous future validation should involve replication in an independent sample.

## Conclusions

In this prognostic study seeking to identify proximal predictors of suicidal thoughts following ED discharge, predictors derived from EMAs yielded the highest predictive accuracy, whereas passive predictors, alone or in combination with EMAs, had negligible predictive accuracy. Strongest predictors of next-day suicidal thoughts were related to prior suicidal ideation and encompassed different timescales. The fact that wearable devices did not outperform self-reporting in this and prior studies points to greater utility of EMAs in predicting short-term suicidal ideation, warranting additional examination in different samples. Additionally, these results have implications for developing decision rules, based on prediction algorithms, that guide the delivery of support in real time to prevent or ameliorate proximal risk for suicidal thoughts.

## Notes

Supplement 1. **eMethods 1.** Ecological Momentary Assessment Items **eMethods 2.** Details on Preparing Fitbit Features **eMethods 3.** Variable Selection Approach Using Penalized GEE **eMethods 4.** Framework for Prediction Modeling Using GLMM Trees **eFigure 1.** Suicidal Ideation Time Series Plots for Each Participant **eFigure 2.** Relative Variable Importance From GLMM Trees Using EMA Data **eFigure 3.** Relative Variable Importance From GLMM Trees Using EMA and Passive Data **eFigure 4.** Relative Variable Importance From GLMM Trees Using EMA Data, Without Suicidal Ideation-Related Features **eTable.** Estimated Odds Ratios, Using Mixed Effects Logistic Regression
eReferences

Supplement 2.
Data Sharing Statement
