# Supplementary material for: Ecological Momentary Assessments and Passive Sensing in the Prediction of Short-Term Suicidal Ideation in Young Adults
Source: JAMA Netw Open. 2023 Aug 8;6(8):e2328005. doi: 10.1001/jamanetworkopen.2023.28005 (PMC10410485; doi:10.1001/jamanetworkopen.2023.28005)
Supplement: Supplement 1. — eMethods 1. Ecological Momentary Assessment Items eMethods 2. Details on Preparing Fitbit Features eMethods 3. Variable Selection Approach Using Penalized GEE eMethods 4. Framework for Prediction Modeling Using GLMM Trees eFigure 1. Suicidal Ideation Time Series Plots for Each Participant eFigure 2. Relative Variable Importance From GLMM Trees Using EMA Data eFigure 3. Relative Variable Importance From GLMM Trees Using EMA and Passive Data eFigure 4. Relative Variable Importance From GLMM Trees Using EMA Data, Without Suicidal Ideation-Related Features eTable. Estimated Odds Ratios, Using Mixed Effects Logistic Regression eReferences [file jamanetwopen-e2328005-s001.pdf]

Supplemental Online Content

Czyz EK, King CA, Al-Dajani N, Zimmermann L, Hong V, Nahum-Shani I. Ecological momentary assessments and passive sensing in the prediction of short-term suicidal ideation in young adults. *JAMA Netw Open.* 2023;6(8):e2328005. doi:10.1001/jamanetworkopen.2023.28005

eMethods 1. Ecological Momentary Assessment Items

eMethods 2. Details on Preparing Fitbit Features

eMethods 3. Variable Selection Approach Using Penalized GEE

eMethods 4. Framework for Prediction Modeling Using GLMM Trees

eFigure 1. Suicidal Ideation Time Series Plots for Each Participant

eFigure 2. Relative Variable Importance From GLMM Trees Using EMA Data

eFigure 3. Relative Variable Importance From GLMM Trees Using EMA and Passive Data

eFigure 4. Relative Variable Importance From GLMM Trees Using EMA Data, Without Suicidal Ideation-Related Features

eTable. Estimated Odds Ratios, Using Mixed Effects Logistic Regression

eReferences

This supplemental material has been provided by the authors to give readers additional information about their work.

© 2023 Czyz EK et al. *JAMA Network Open.*

eMethods 1. Ecological momentary assessment items

Variable (Range)	Source	Prompt
Happy (1-5)	EMAs (4x per day)	Please rate how much you felt this way in the last hour: Happy
Miserable (1-5)	EMAs (4x per day)	Please rate how much you felt this way in the last hour: Miserable
Angry (1-5)	EMAs (4x per day)	Please rate how much you felt this way in the last hour: Angry
Nervous (1-5)	EMAs (4x per day)	Please rate how much you felt this way in the last hour: Nervous
Sad (1-5)	EMAs (4x per day)	Please rate how much you felt this way in the last hour: Sad
Rumination (1-7)	EMAs (4x per day)	Please rate how much these fit your experience in the last hour:<br>I was dwelling on my feelings and problems
Worry (1-7)	EMAs (4x per day)	Please rate how much these fit your experience in the last hour:<br>I was worried about things that could happen
Agitation (1-7)	EMAs (4x per day)	Please rate how much these fit your experience in the last hour:<br>I felt so stirred up inside that I wanted to scream
Hopelessness (1-4)	EMAs (4x per day)	Please rate how much you felt this way in the last hour: I see only bad things ahead of me, not good things
Buren (1-7)	EMAs (4x per day)	Please rate how much these fit your experience in the last hour: I felt people in my life would be happier without me
Close to others (1-7)	EMAs (4x per day)	Please rate how much these fit your experience in the last hour: I felt close to my family
Self-efficacy (0-10)	EMAs (4x per day)	How confident are you that you will be able to keep yourself from attempting suicide?
Duration of death thoughts (0-4)	EMAs (4x per day)	In the last hour, did you wish you were dead or that you could go to sleep and not wake up? How long did these thoughts last?
Duration of suicidal ideation (0-4)	EMAs (4x per day)	In the last hour, did you have thoughts of killing yourself? How long did these thoughts last?
Intensity of suicidal ideation (0-5)	EMAs (4x per day)	In the last hour, how strong was your intention to kill yourself?

© 2023 Czyz EK et al. *JAMA Network Open.*

Variable (Range)	Source	Prompt
Frequency of death thoughts (0-4)	Evening EMA survey	Today, how many times did you wish you were dead or that you could go to sleep and not wake up?
Frequency of suicidal ideation (0-4)	Evening EMA survey	Today, how many times did you have thoughts of killing yourself?
Intensity of suicidal ideation (0-5)	Evening EMA survey	Today, how strong was your intention to kill yourself?
Negative relationship events (dichotomized)	Evening EMA survey	Today, did you have a negative relationship event such as a serious or disruptive argument, separation, or falling out with someone? [includes 6 relationship categories: romantic; friend/peer; teacher/boos; parent; non-parent relative; other)
Coping (0-6)	Evening EMA survey	Today, how much did you do these things to deal or cope with your feelings or any stressful situations? [includes cognitive, non-cognitive, and support-seeking strategies]
Sleep quality (0-4)	Morning EMA survey	How would you rate the quality of your sleep?
Nonsuicidal self-injury (dichotomized)	Morning EMA survey	Thinking about yesterday how many times did you harm yourself or hurt your body on purpose (such as cutting, burning, biting, hitting self) without the intention to die
Alcohol (in standard drinks)	Morning EMA survey	How many standard drinks containing alcohol did you have yesterday?<br>By a standard drink we mean a 12 ounce can or glass of beer or wine cooler; or a 5-ounce glass of wine, or a drink with 1 shot of liquor.

© 2023 Czyz EK et al. *JAMA Network Open.*

eMethods 2. Details on preparing Fitbit features

Item Name	Item Values/Description	General Description
RestingHeartRate	Integer, observed range:<br>50-105 bpm	Raw variable from daily Fitabase totals.<br>"Resting heart rate value." [from Fitabase]
TotalMinutesAsleep	Integer, observed range:<br>0-32000 steps	Raw variable from daily Fitabase totals.<br>"Total number of steps taken." [from Fitabase]
Adherence	Continuous proportion, range:<br>0-1	Daily proportion of total minutes with a heart rate value (i.e., Value not missing nor zero) divided by 1440 (24 hours * 60 min) minutes in a day.<br>(Input variable: Value from 1 Min HR [from Fitabase])
Num_adherence	Continuous, range:<br>0-1440 minutes	Daily number of minutes with a heart rate value (i.e., Value not missing nor zero). Intended to indicate wear-time.<br>(Input variable: Value from 1 Min HR [from Fitabase])
Denom_adherence	Fixed: 1440 minutes	1440 (24 hours * 60 min) minutes in a day
TotalSteps_corrected	Discrete, observed range:<br>0-32000 steps	Daily number of steps where values with TotalSteps equal to zero and Adherence equal to zero are set to be a missing value.<br>(Input variables: TotalSteps [from Fitabase] and Adherence [computed, as detailed above])
RMSSD_mean	Continuous, observed range:<br>0-169.51 ms	Daily average of root mean squared value of the successive differences of R-R intervals, approximated using 1 minute pulse rates. The average of the successive differences is computed over 5-minute intervals and subsequently averaged over the 24-hour day.<br>(Input variables: RR interval [calculation, as detailed below])
RR interval	Continuous, observed range:<br>250-1700 ms	The R-R interval is defined as the time in milliseconds between consecutive heart beats. In other words, the R-R interval is estimated by the inverse of the pulse rate. We convert the heart rate (in beats per minute) to the R-R interval (in milliseconds) as follows:<br>$RR = \frac{1\ min}{HR\ beats} \times \frac{60\ sec}{1\ min} \times \frac{1000\ ms}{1\ sec}$<br>(Input variables: Value from 1 Min HR [from Fitabase])

© 2023 Czyz EK et al. *JAMA Network Open.*

eMethods 3. Variable selection approach using penalized GEE

The present study performed variable selection to identify features correlated with the outcome of next-day suicidal ideation, using penalized generalized estimating equations (PGEE) developed by Wang and collegues.[1] We opted to use PGEE regularization, as the present study concerned intensive longitudinal data with a large number of features,[1] and as this approach was previously used in mobile health research utilizing repeated measurements.[2] The PGEE package in R was used,[3] and code files are available via github.com/lzimmermann4/Short-term_SI.

Below, we describe the parameters used in the estimation algorithm for the tuning parameter. First, the cross-validated value of the tuning parameter (λ) with a grid search was obtained. That is, Wang and colleagues' penalized GEE[1] incorporates a SCAD penalty, which involves parameters λ and a. In the PGEE R package, Inan and Wang[3] specify $a = 3.7$ as recommended.[4] Further details on properties and functional form of the SCAD penalty are detailed in Fan and Li.[4] For deriving the optimal λ, namely the value that minimizes the cross-validated prediction error, 5-fold cross-validation was employed over a grid with an epsilon threshold of $10,^{-6}$ maximum iterations of 30, and tolerance of $10,^{-3}$ as conventionally specified.[3] This was followed by employing PGEE with a first order autoregressive correlation structure, binomial family for the outcome, and the optimal λ. Wang and colleagues[1] demonstrated the robustness of PGEE to misspecification of the correlation structure.

© 2023 Czyz EK et al. *JAMA Network Open.*

eMethods 4. Framework for prediction modeling using GLMM trees

We applied multi-level classification and regression tree (CART) models to predict next-day suicidal ideation. Designed to accommodate multi-level and longitudinal data structures, these CART models employ the generalized linear mixed model (GLMM) tree method developed by Fokkema and colleagues.[5,6] We used the glmertree R package[6] to apply this method. In this approach, an algorithm akin to expectation maximization is used to iteratively estimate the random effects from a GLMM, wherein we specified a random intercept model, and estimate the fixed effects from the tree, assuming that the random effects are known. This procedure is common to longitudinal tree-based methods that incorporate mixed effects.[7-9]

We used a variance-based method for computing the variable importance (VI) scores for included predictors,[10] developed by Greenwell and colleagues.[10] The *vip* package in R was used to obtain VI scores.[11] The variable importance was averaged across 5 folds in the k-fold cross-validation and 10 repetitions.

© 2023 Czyz EK et al. *JAMA Network Open.*

eFigure 1. Suicidal ideation time series plots for each participant

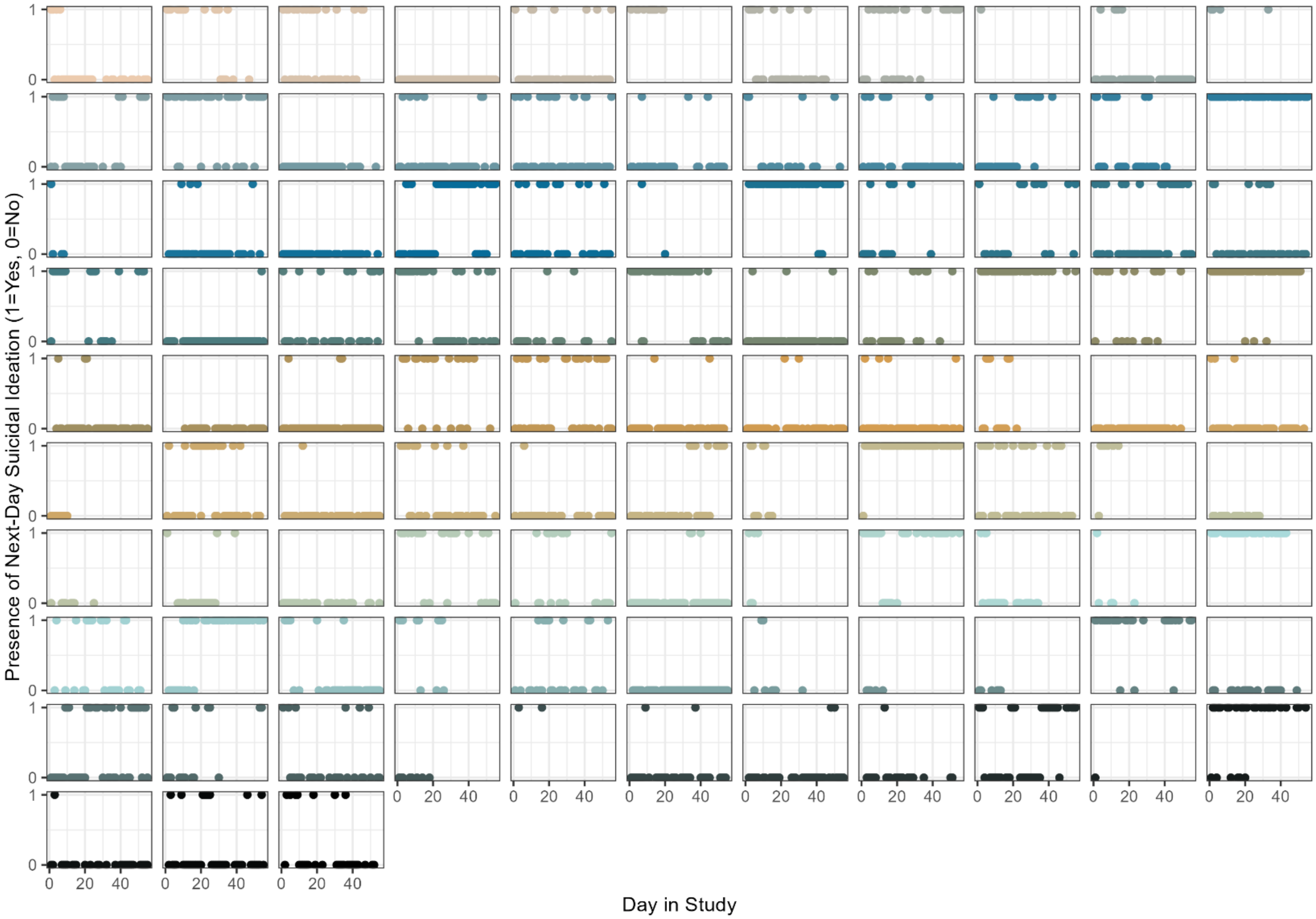

eFigure 1. Presence of Next-Day Suicidal Ideation (SI) for each participant, across the 8-week study period (N=102 participants). Note that eligible study participants were young adults recruited from the emergency department, with a last-month suicide attempt and/or last-week suicidal ideation, and who met exclusion criteria.

© 2023 Czyz EK et al. *JAMA Network Open.*

eFigure 2. Relative variable importance from GLMM trees using EMA data

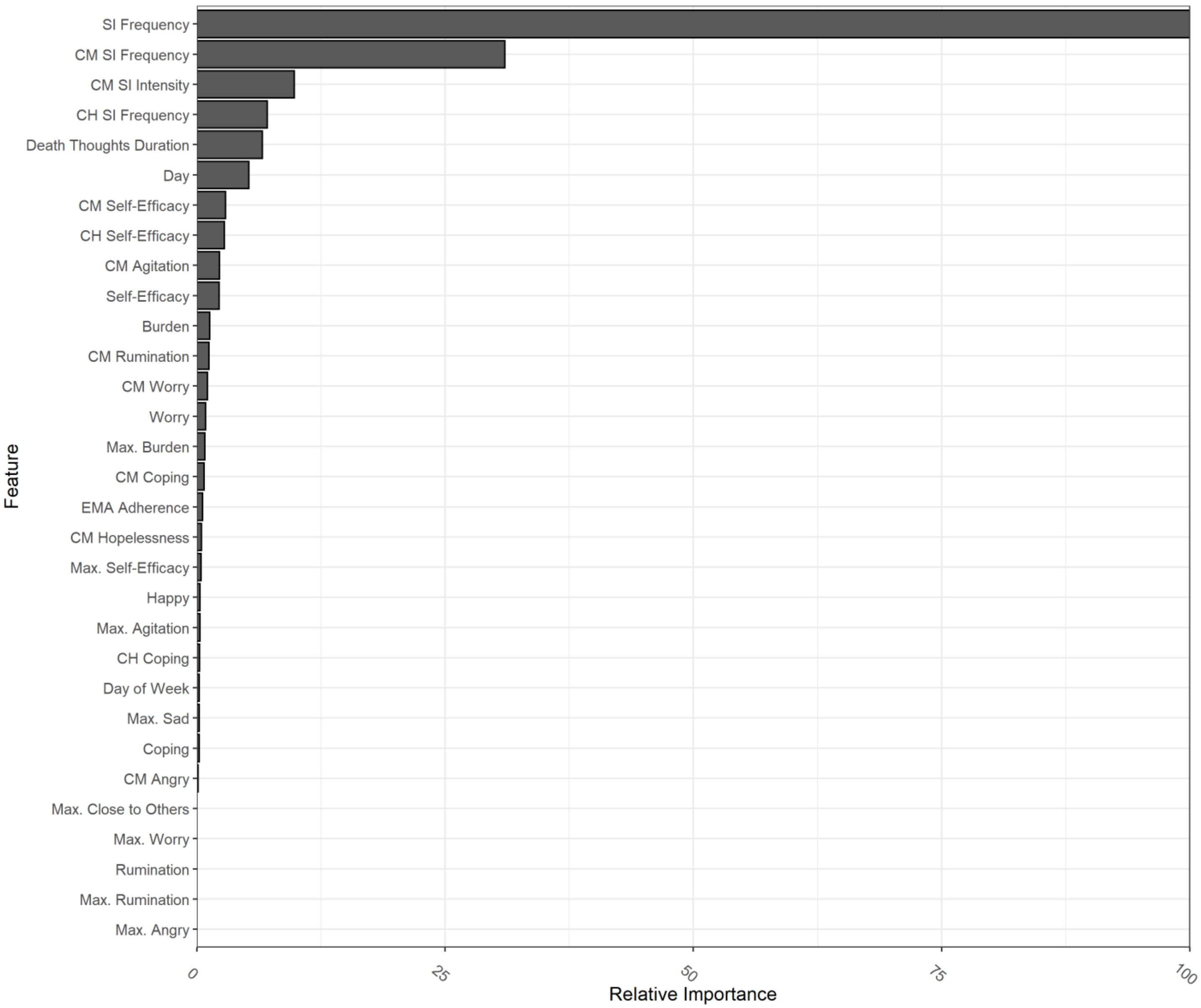

eFigure 2. Variable importance from EMA model predicting Next-Day Suicidal Ideation (SI), using GLMM trees, averaged across 5-fold CV and 10 repetitions (N=3,126). Note that CM=cumulative mean, CH=change from cumulative mean, Max.= maximum score from within-day EMAs.

© 2023 Czyz EK et al. *JAMA Network Open.*

eFigure 3. Relative variable importance from GLMM trees using EMA and passive data

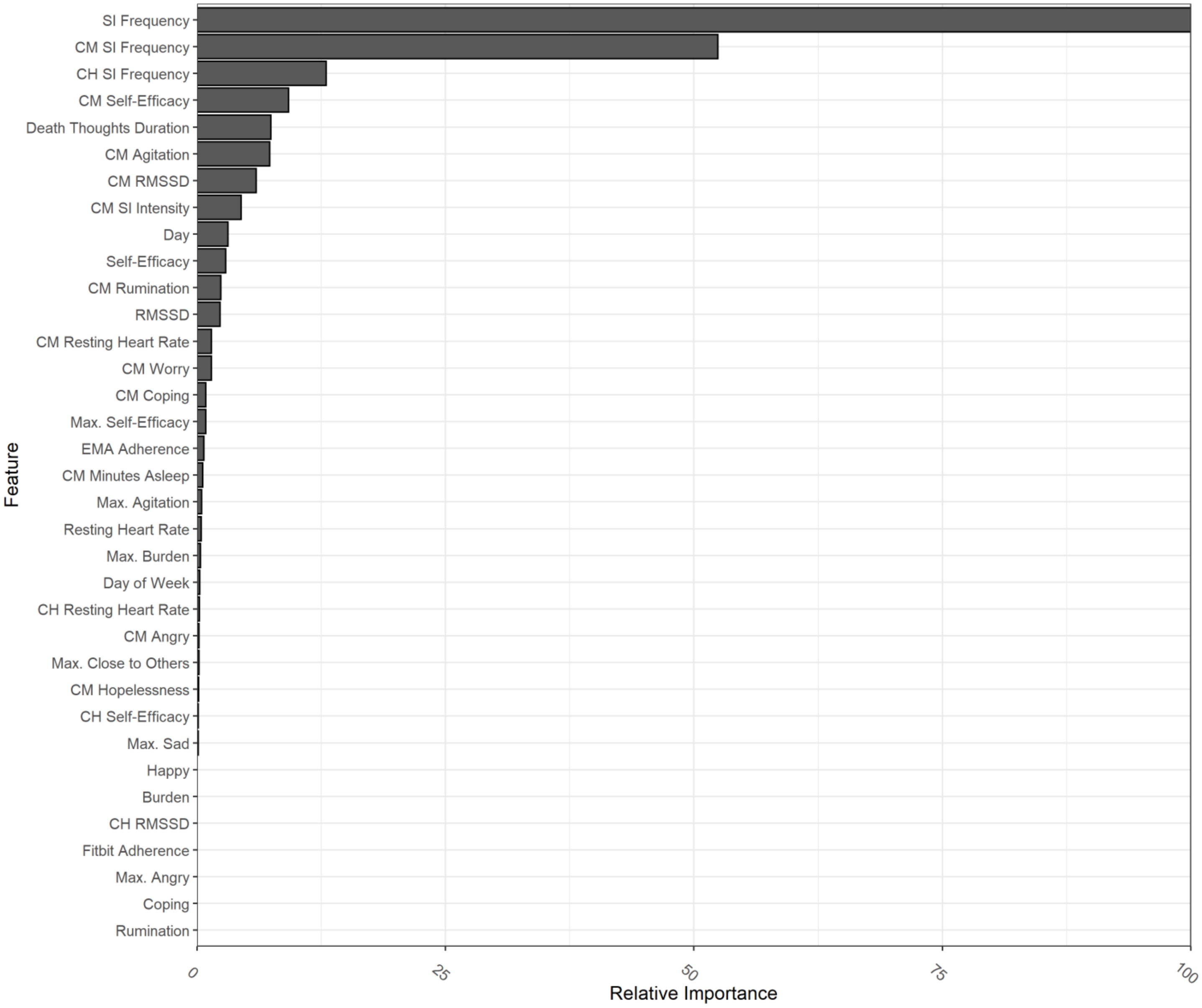

eFigure 3. Variable importance from EMA and passive model predicting Next-Day Suicidal Ideation (SI), using GLMM trees, averaged across 5-fold CV and 10 repetitions (N=1,804). Note that CM=cumulative mean, CH=change from cumulative mean, Max.=maximum over 4 within-day EMAs.

© 2023 Czyz EK et al. *JAMA Network Open.*

eFigure 4. Relative variable importance from GLMM trees using EMA data, without suicidal ideation-related features

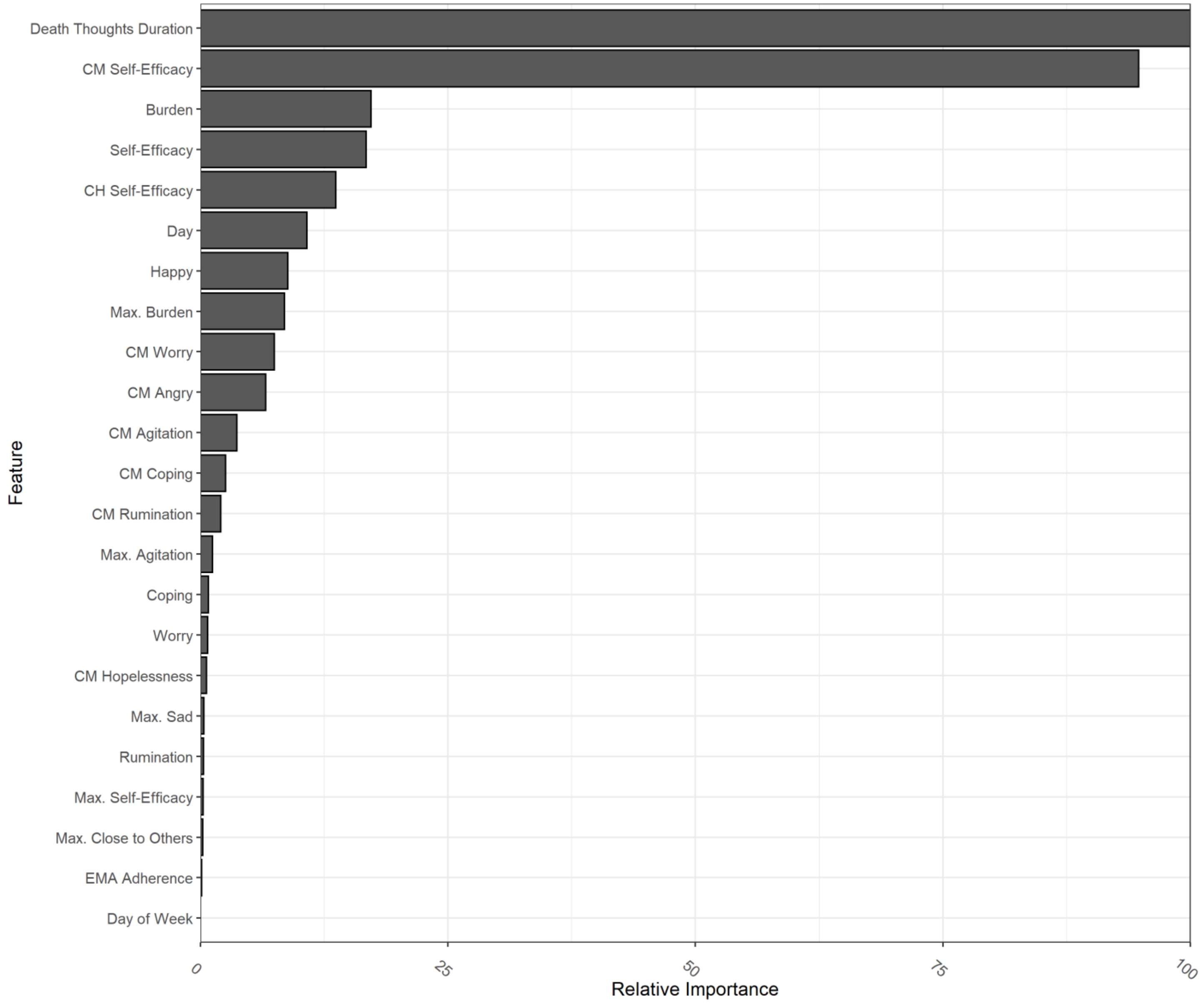

eFigure 4. Variable importance from EMA model predicting Next-Day Suicidal Ideation (SI), without suicidal ideation-related features, using GLMM trees, averaged across 5-fold CV and 10 repetitions (N=3,126). Note that CM=cumulative mean, CH=change from cumulative mean, Max.= maximum score from within-day EMAs.

© 2023 Czyz EK et al. *JAMA Network Open.*

eTable. Estimated odds ratios (ORs), using mixed effects logistic regressions

Source	Covariate	OR (95% Confidence Interval)	N Obs.
EMAs (4x per day)	Happy	0.664 (0.573, 0.768)	3,126
	CM Angry	2.267 (1.415, 3.633)	3,126
	Max. Angry	1.316 (1.192, 1.454)	3,126
	Max. Sad	1.363 (1.244, 1.494)	3,126
	Rumination	1.314 (1.213, 1.424)	3,126
	CM Rumination	1.149 (0.964, 1.371)	3,126
	Max. Rumination	1.202 (1.129, 1.279)	3,126
	Worry	1.305 (1.203, 1.416)	3,126
	CM Worry	1.343 (1.124, 1.603)	3,126
	Max. Worry	1.203 (1.129, 1.282)	3,126
	CM Agitation	1.149 (0.934, 1.415)	3,126
	Max. Agitation	1.183 (1.115, 1.255)	3,126
	CM Hopelessness	2.591 (1.662, 4.037)	3,126
	Burden	1.696 (1.528, 1.883)	3,126
	Max. Burden	1.437 (1.334, 1.549)	3,126
	Max. Close to Others	0.849 (0.787, 0.916)	3,126
	Self-Efficacy	0.693 (0.637, 0.754)	3,126
	CM Self-Efficacy	0.611 (0.521, 0.717)	3,126
	CH Self-Efficacy	0.686 (0.618, 0.761)	3,126
	Max. Self-Efficacy	0.755 (0.688, 0.828)	3,126
	Death Thoughts Duration	4.172 (3.318, 5.246)	3,126
Evening EMA Survey	CM SI Intensity	17.149 (10.456, 28.127)	3,126
	SI Frequency	3.329 (2.858, 3.878)	3,126
	CM SI Frequency	9.654 (6.968, 13.375)	3,126
	CH SI Frequency	2.985 (2.545, 3.502)	3,126
	Coping	0.997 (0.925, 1.074)	3,126
	CM Coping	0.914 (0.753, 1.109)	3,126
	CH Coping	1.103 (0.931, 1.102)	3,126
Fitbit (Daily)	Resting Heart Rate	1.009 (0.977, 1.042)	2,177
	CM Resting Heart Rate	1.021 (0.978, 1.065)	2,177
	CH Resting Heart Rate	0.995 (0.948, 1.043)	2,177
	RMSSD	0.995 (0.970, 1.020)	2,177
	CM RMSSD	0.945 (0.900, 0.992)	2,177
	CH RMSSD	1.015 (0.985, 1.046)	2,177
	CM Minutes Asleep	0.999 (0.996, 1.002)	2,177

Notes: EMA=ecological momentary assessment, CM=cumulative mean, CH=change from cumulative mean, Max.=maximum score from within-day EMAs, SI=suicidal ideation, RMSSD=root mean square of successive difference from heart rate.
Coping reflects the sum of three coping types reframe, talk, and distract.
Results reflect specification of a random intercept and adjusting for day in study (1-56), day of week (1-7) and missingness indicator. Covariates are mean-centered.

© 2023 Czyz EK et al. *JAMA Network Open.*

eReferences

1. Wang L, Zhou J, Qu A. Penalized generalized estimating equations for high-dimensional longitudinal data analysis. *Biometrics*. 2012;68(2):353-360.

2. Inan G, Wang L. PGEE: An R package for analysis of longitudinal data with high-dimensional covariates. *R J*. 2017;9(1):393.

3. Fan J, Li R. Variable selection via nonconcave penalized likelihood and its oracle properties. *J Am Stat Assoc*. 2001;96(456):1348-1360.

4. Sauerbrei W. The use of resampling methods to simplify regression models in medical statistics. *J R Stat Soc Ser C Appl Stat*. 1999;48(3):313-329.

5. Fokkema M, Edbrooke-Childs J, Wolpert M. Generalized linear mixed-model (GLMM) trees: A flexible decision-tree method for multilevel and longitudinal data. *Psychother Res*. 2020:1-13.

6. Fokkema M, Smits N, Zeileis A, Hothorn T, Kelderman H. Detecting treatment-subgroup interactions in clustered data with generalized linear mixed-effects model trees. *Behav Res Methods*. 2018;50:2016-2034.

7. Hajjem A, Larocque D, Bellavance F. Generalized mixed effects regression trees. *Stat Probab Lett*. 2017;126:114-118.

8. Sela RJ, Simonoff JS. RE-EM trees: A data mining approach for longitudinal and clustered data. *Mach Learning*. 2012;86:169-207.

9. Ngufor C, Van Houten H, Caffo BS, Shah ND, McCoy RG. Mixed effect machine learning: A framework for predicting longitudinal change in hemoglobin A1c. *J Biomed Inform*. 2019;89:56-67.

10. Greenwell BM, Boehmke BC, McCarthy AJ. A simple and effective model-based variable importance measure. *arXiv preprint arXiv:1805.04755*. 2018.

11. Greenwell B, Boehmke B, Gray B. Package 'vip'. *Variable Importance Plots*. 2020;12(1):343-366.

© 2023 Czyz EK et al. *JAMA Network Open.*
