# Supplementary material for: Ecological Momentary Assessments and Passive Sensing in the Prediction of Short-Term Suicidal Ideation in Young Adults
Source: JAMA Netw Open. 2023 Aug 8;6(8):e2328005. doi: 10.1001/jamanetworkopen.2023.28005 (PMC10410485; doi:10.1001/jamanetworkopen.2023.28005)
Supplement: Supplement 2. — Data Sharing Statement [file jamanetwopen-e2328005-s002.pdf]

Data Sharing Statement

Czyz. Ecological Momentary Assessments and Passive Sensing in the Prediction of Short-Term Suicidal Ideation in Young Adults. *JAMA Netw Open*. Published August 08, 2023. doi:10.1001/jamanetworkopen.2023.28005

Data
Data available: Yes
Data types: Deidentified participant data
How to access data: The data that support the findings of this study are available from the corresponding author upon reasonable request.
When available: With publication

Supporting Documents
Document types: Statistical/analytic code
How to access documents: Analytic code will be available via github repository listed in the manuscript.
When available: With publication

Additional Information
Who can access the data: Data will be made available for researchers whose proposed use of the data has been approved.
Types of analyses: Data will be available for a specified purpose.
Mechanisms of data availability: Data will be made available with a signed data access agreement.
